# Analysis of Climate Change Affecting German Forests by Combination of Meteorological and Phenological Data within a GIS Environment

**DOI:** 10.1100/tsw.2007.15

**Published:** 2007-03-21

**Authors:** Winfried Schröder, Roland Pesch, Gunther Schmidt, Cordula Englert

**Affiliations:** Department of Landscape Ecology, University of Vechta, PO 1553, D-49356 Vechta, Germany

**Keywords:** air temperature, biomonitoring, climate change, data compilation, geostatistics, plant phenology

## Abstract

The regional assessment of global change effects on plant phenology usually relies on local observations that need to be up-scaled. Therefore, methodological difficulties mostly related to data spatial resolution and congruency arise while performing broader-scale evaluations. Geostatiscs could be a useful tool to solve this type of problem, provided that a database with adequate spatial and temporal resolution is available. An assessment of variations in air temperature and plant phenology was carried out at the country level by using two German datasets regarding spring phenological phases of 15 plant species and air temperature. The data were collected from 1961–2002 at 1,279 and 675 sites, respectively. The annual mean air temperature in Germany was found to rise from 8.3°C in the 1961–1990 period to 9.1°C in the 1991–2002 term. The overall 15-species mean for the start of spring was found to be 6 days earlier in the latter period. The geostatistical analysis of the data revealed the suitability of* Syringa vulgaris* to be used as an indicator species to detect phenological changes in German forests. Moreover, their spatial patterns were found to be related to altitude and latitude. Therefore, geostatistics proved to be a useful tool to overcome some of the methodological problems related to the regional assessments of global change impacts on terrestrial ecosystems.

## GOALS AND BACKGROUND

The extent and the rate of current climate change exceed the natural variation during the last thousand years. The European Environment Agency[1] identified 22 climate change state and impact indicators, including air temperature and plant phenology. The global annual mean temperature has increased by about 0.7°C, whereas the European mean rose by 0.95°C in the last hundred years. From 1990–2100, the air temperatures are expected to increase by 1.4–5.8°C globally and by 2.0–6.3°C in Europe. These changes have altered plant performance and are expected to do so in the future. For instance, the average annual growing season in Europe was extended by about 10 days between 1962 and 1995, and greenness of vegetation as a measure of plant productivity increased by 12%. This tendency is assumed to be balanced by an increased risk of water shortage in central and southern Europe.

Significant ecophysiological effects of climate change on plant species, phytocoenosis, and terrestrial ecosystems have been found from studies performed at the local scale[2,3,4,5]. The use of such local findings assumes that the phenological data they are related to are also valid at a regional scale[6]. Climate changes and trends in plant phenology have been analyzed at the European scale[7,8,9] and for the territory of Germany[10,11]. At any scale, a significant shift towards an earlier occurrence of spring phenological phases could be corroborated.

Though several parameters related to environmental change have the potential to influence the timing of flowering and other developmental stages, the shifting of plant phenology across the northern hemisphere has been mostly interpreted as a response to warming[12]. However, sometimes it is difficult to differentiate the effects of two co-occurring variables; for instance, a strong correlation between N deposition and air temperature was observed in Finland and Sweden, and made it difficult to distinguish the effects of one variable from the other[13,14]. This would also apply to factors related to air temperature profiles, such as latitude, altitude, and some topographical features. Thus, there is a need to monitor and map plant phenological variations as related to seasonal changes of environmental conditions, particularly air temperature, precipitation, and soil moisture[15,16,17,18].

While success in modeling phenology at the species level has been achieved, the prediction of regional plant phenological variations related to climate change is still a problem. This is mainly due to the fact that phenological data are often collected apart from the sites where meteorological and any other relevant environmental parameters are recorded[19,20,21], as the extrapolation of values obtained at the local scale to broader scales is the precondition for mapping and modeling plant phenological changes occurring at the regional level[22,23,24,25]. Geostatistical approaches could be useful to solve this type of difficulty, provided an adequate spatial and temporal data resolution is available.

In this paper, we used the database collected in Germany involving the records of air temperature and phenological traits of 15 plant species to assess preliminarily whether temporal and spatial trends can be identified, and to evaluate the relationships between both types of data.

## METHODS

### Air Temperature and Plant Phenology Datasets

The German Meteorological Service (Deutscher Wetterdienst, DWD) provides air temperature data recorded across Germany at 675 sites, according to standardized methods[26]. Also, plant phenology is monitored in Germany away from those sites by the DWD, following two complementary approaches: (1) since the middle of the 1950s, the phenology of cloned plants growing in Germany's 25 International Phenological Gardens (IPG) has been observed; (2) since 1936, the phenology of plants has been monitored at up to 6,400 sites from all over Germany following a standard protocol[27]. Accordingly, the phenological data are collected at every site with a frequency of 2 or 3 days/week. The monitored plants must be representative of their respective areas. The altitude of the phenological monitoring sites must match the mean altitude of the area by ±50 m. The plants to be monitored must be healthy and should not grow in deep hollows and narrow valleys, or on slopes that are exposed to the south or to the north. The selected plant species at a given site must remain in the same plots in the long term. The phenological network provides data on 269 plant species with a dense geographical and ecological coverage of Germany.

We concentrated on four phenological phases monitored at up to 1,279 sites with regard to 15 species: blooming (B), leaf unfolding (L), ear formation (E), and sprouting in May (S), which have been reported to be valuable indicators of the initiation of springtime[27] — *Achras* (B), *Aesculus hippocastanum* (L, B), *Anemone nemorosa* (B), *Betula pendula* (L), *Crataegus laevigata* (B), *Galanthus nivalis* (B), *Picea pungens* (S), *Prunus avium* (B), *Quercus robur* (L), *Ribes rubrum* (B), *R. uva-crispa* (B), *Salix caprea* (B), *Secale cereale* (E), *Syringa vulgaris* (B), *Taraxum officinale* (B). The beginning of the phases is indicated as "days after the beginning of the calendar year".

## Trend Analysis of Phenological and Air Temperature Data

The data involving the onset of the above-mentioned spring phases and plant species (Table 1) from up to 1,279 monitoring sites and the air temperature monthly mean averages from 675 meteorological stations were compiled. Two time intervals of equal length were considered, 1961–1990 and 1991–2002, to evaluate whether significant changes have occurred in regards to both air temperature and the onset of spring plant phenological phases. Annual air temperature averages for both periods were calculated. We excluded the phenological data from those monitoring sites showing less than 10% of all observations within the respective monitoring interval, i.e., 27 (1961–1990) or 8 (1991–2002) records of each phenological phase, respectively. The Mann-Whitney U test was used to determine whether the selected phenological traits were significantly different in both periods; alpha was set at 0.05 for all comparisons.

**TABLE 1**
**Statistical Analyses of the Variations in the Start of the Spring Phases of 15 Plant Species in Germany Between the 1961–1990 and 1991–2002 Periods**

| Plant | Phase | No. of Sites (1961–1990) | No. of Sites (1991–2002) | Min (days) | Max (days) | Mean (days) | St.dev. (days) | Diff. signif.? |
|---|---|---|---|---|---|---|---|---|
| *Achras* | B | 932 | 604 | –27.8 | 8.9 | –7.2 | 3.6 | $p < 0.0001$ |
| *Aesculus hippocastanum* | L | 1,146 | 1,067 | –27.2 | 35.1 | –5.7 | 4.6 | $p < 0.0001$ |
| | B | 1,161 | 959 | –20.5 | 80.3 | –4.6 | 4.2 | $p < 0.0001$ |
| *Anemone nemorosa* | B | 897 | 920 | –39.8 | 29.9 | –6.8 | 6.4 | $p < 0.0001$ |
| *Betula pendula* | L | 1,092 | 1,056 | –24.9 | 13.1 | –5.3 | 4.0 | $p < 0.0001$ |
| *Crataegus laevigata* | B | 884 | 735 | –57.6 | 19.0 | –7.1 | 5.1 | $p < 0.0001$ |
| *Galanthus nivalis* | B | 1,279 | 1,195 | –38.3 | 30.2 | –10.1 | 6.5 | $p < 0.0001$ |
| *Picea pungens* | S | 728 | 837 | –29.8 | 11.4 | –6.1 | 4.6 | $p < 0.0001$ |
| *Prunus avium* | B | 960 | 545 | –28.5 | 10.1 | –5.5 | 3.4 | $p < 0.0001$ |
| *Quercus robur* | L | 914 | 799 | –29.4 | 14.6 | –5.6 | 4.3 | $p < 0.0001$ |
| *Ribes rubrum* | B | 914 | 647 | –22.6 | 28.8 | –2.7 | 4.6 | $p < 0.0001$ |
| *R. uva-crispa* | B | 949 | 786 | –31.3 | 29.3 | –6.5 | 4.6 | $p < 0.0001$ |
| *Salix caprea* | B | 1,169 | 999 | –51.8 | 26.8 | –8.3 | 6.1 | $p < 0.0001$ |
| *Secale cereale* | E | 713 | 423 | –29.4 | 19.9 | –7.2 | 4.1 | $p < 0.0001$ |
| *Syringa vulgaris* | B | 1,222 | 1,020 | –22.3 | 25.1 | –6.2 | 3.5 | $p < 0.0001$ |
| *Taraxacum officinale* | B | 1,079 | 1,143 | –33.7 | 15.1 | –6.0 | 4.9 | $p < 0.0001$ |

Phase: B = beginning of flowering; L = unfolding of leaves; E = beginning of ear formation; S = sprouting in May.

## Data Integration, Analysis, and Geostatistical Estimation

Since the meteorological and phenological networks did not match spatially, surface estimation methods were needed to ensure a valid spatial correlation of both datasets. Geostatistics enable us to analyze and model the spatial autocorrelation of biological, chemical, and physical measurements and, based on this variographic modeling, to calculate surface estimations by spatially weighted kriging interpolation. The quality of estimation can be proved by cross-validations: Each measurement is sequentially extracted from the sample and it is subsequently estimated by kriging according to the variographic model. The differences between the measured and geostatistically estimated values can then be quantified[28]. This approach enables us to correlate measurement data of incongruent monitoring networks, as kriging provides an estimate on the measurement of the complementary monitoring network for each site-specific measurement value. Thus, in this investigation, each site-specific meteorological measurement was correlated with a site-specific phenological estimate and, vice versa, each meteorological estimate was spatially related to a phenological measurement.

The raw data from the meteorological and the phenological networks, the related surface estimations, and a map on land coverage (CORINE 2000)[29] were integrated into a GIS (ArcView GIS 3.3). From the land cover map, the forested areas were extracted and linked to the phenological monitoring sites.

## RESULTS AND DISCUSSION

The data presented in Table 1 indicate the earlier occurrence (number of days from the start of the year) of spring phases under investigation, as well as the range of variation for the 15 species involved in the study and their overall average. On average, the onset of plant spring phases occurred 6 days earlier in the 1991–2002 period when compared to the 1961–1990 interval.

The spring phases of the 15 plant species appeared to be related to air temperature records. As can be seen in Fig. 1 (left), the long-term annual mean rose from 8.3°C (1961–1990) to 9.1°C (1991–2002). The highest temperatures were measured along the Upper and Lower Rhine Valley in the western part of Germany, and in the lowlands of northern Germany. The highest increases were recorded in the low mountain ranges in central and southeastern Germany.

The examined phenological phases of forest trees did not reveal a clear spatial pattern and, thus, no surface estimations were calculated in this investigation. Future studies should test whether alternative geostatistical methods like co-kriging or universal kriging will prove satisfactory, and so enable valid surface estimations for forest trees. Furthermore, the most frequent forest tree species in Germany, *Picea abies*, *Fagus sylvatica*, and *Pinus sylvestris*, should be involved in those analyses.

However, *Syringa vulgaris* (common lilac) was proposed as an indicator of potential phenological effects of global warming in forested areas, as it was monitored at 98% of the phenological sites located in forests and showed the highest spatial autocorrelation in comparison to all the species mentioned in Table 1. Also, this species matched the overall mean of the air-temperature shifts of the examined spring phases of the 15 plant species involved in this study (see Table 1) showing a low standard deviation.

Fig. 1 (middle) shows the shift of the blooming of *S. vulgaris* in Germany. The beginning of flowering was mapped for two time periods (1961–1990 and 1991–2002) based on observations made at 1,222 (1961–1990) and 1,020 (1991–2002) locations, respectively; 637 and 311, respectively, of these monitoring sites were situated in forested areas.

On average, the blooming of *S. vulgaris* at all sites began 134 days after New Year's Day during the 30-year period, whereas in the 1990s, the flowering started after 128 days. The beginning of blooming revealed similar patterns in the forested sites when compared with nonforested sites, with average values for the 1961–1990 and 1991–2002 terms of 136 days and 129.5 days, respectively. Thus, during the last 12 years, the blooming of *S. vulgaris* occurred 6 and 7.5 days earlier at all sites and inside forests, respectively, than during the former period. The spatial patterns of the onset of the flowering of this species were strongly correlated with the spatial structure of air temperature. Accordingly, the shifting of spring time towards the beginning of the year was less pronounced in the coastal zones in northern Germany and the highlands than in the areas along the Rhine river and the lowlands (Fig. 1), suggesting the existence of latitudinal and altitudinal gradients.

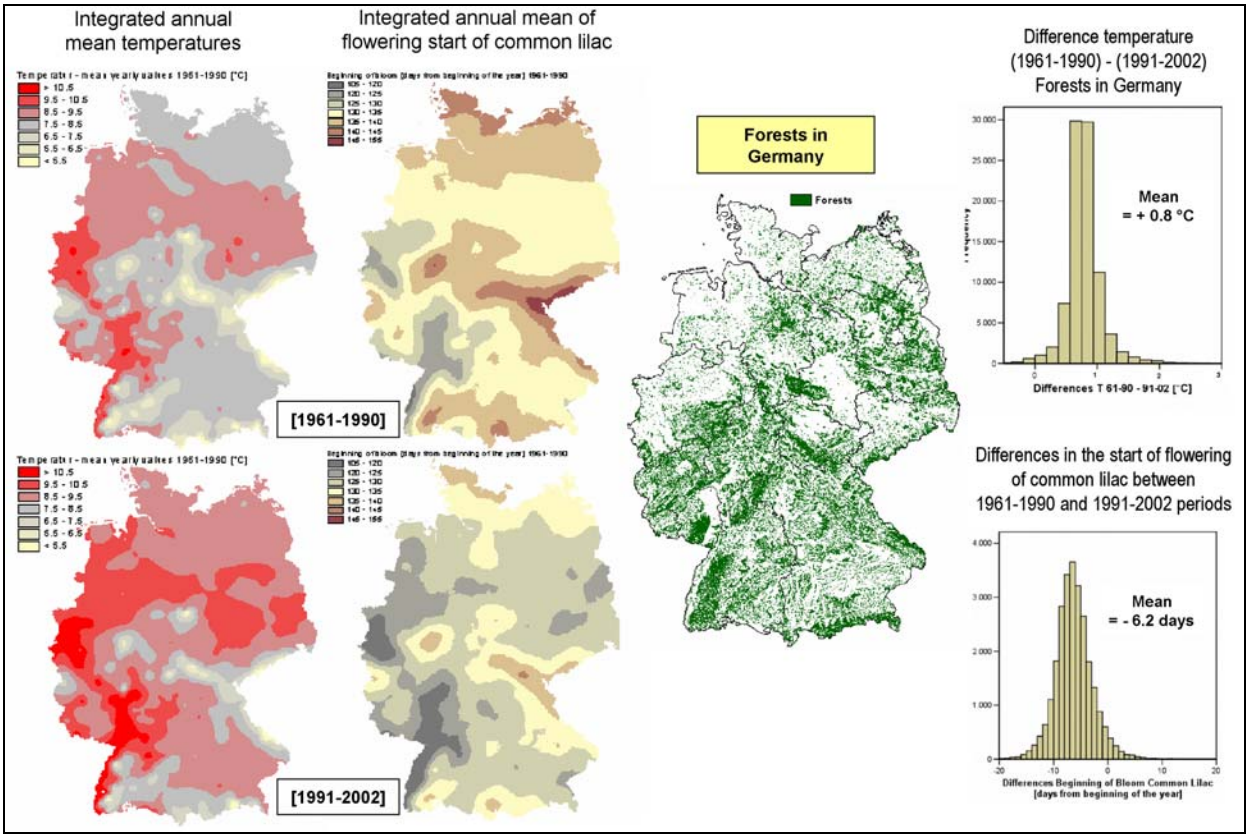

**FIGURE 1.** Shift in air temperature and the beginning of flowering of *S. vulgaris* in Germany 1961–1990 and 1991–2002.

The high quality of the German databases of phenological traits and air temperature, and the geostatistical approach followed in our study proved to solve adequately some of the methodological problems previously detected regarding the regional assessment of global change impact on plant phenology. However, other issues should be considered in the future when assessing the regional implications of climate change on plant phenology. For instance, Badeck et al.[30] found the differences in trend estimates to be caused by the disparity of the relative number of observations in higher and lower latitudes, the involvement of different taxa or groups of phases, and the varying length of the time series analyzed. Moreover, global change encompasses more parameters than just air temperature and their regional patterns should be taken into account, avoiding the usage of their global-scaled averages. In addition, the relationship between climate change and phenological traits other than spring phases should be taken into account.

In our future research, we will focus on these issues as well on scaling-related matters to explain more precisely the regional and temporal variations of plant phenology and their related ecological consequences. Particularly, we will examine the relationships between tree phenology and forest vitality by means of multivariate statistics and dynamic modeling, considering processes such as N-related ecosystem dynamics.
